# Investigating the potential role of innovation and clean energy in mitigating the ecological footprint in N11 countries

**DOI:** 10.1007/s11356-021-18477-0

**Published:** 2022-01-12

**Authors:** Menna Sherif, Dalia M. Ibrahiem, Khadiga M. El-Aasar

**Affiliations:** Faculty of Economics and Political Science, Cairo University, Giza, Egypt

**Keywords:** Ecological footprint, Clean energy, Technological innovation, Panel analysis

## Abstract

This paper seeks to explore the potential function of technological innovation and clean power in mitigating the ecological footprint in the N-11 nations during the phase 1992–2015 by applying panel cointegration analysis. The outcomes of the panel cointegration test signify the occurrence of a long-run relation among the clean energy (CE) variable, the ecological footprint (EF) variable, the per capita GDP (Y) variable, the financial development (FIN) variable, and technological innovation (TI) variable. The outcomes of the VECM signify a long-run causal relation from the ecological footprint (EF) variable to the clean energy (CE) variable, the GDP per capita (Y) variable, and technological innovation (TI) variable. This implies that the environmental degradation faced by the N-11 countries leads to shifting toward clean energy sources and technological innovation in the long run. Thus, the N-11 countries are in need to design policies that enhance shifting toward environmentally friendly energy sources.

## Introduction

Industrialization has been one of the main reasons of climate alteration during the past decades. The industrial revolution that spanned in the eighteenth century has been associated with intensive use of accumulated capital which accelerated the economic growth rates; however, it was also associated with the spurring of harmful gases that were the main causes of global warming and environmental degradation. As a result, the issue of climate change has gained great concerns on the international levels and policy makers started to search for environmentally friendly technologies that could be utilized to maintain the goodness of the environment (Demir et al. 2020). For instance, a report is published annually by the Intergovernmental Panel on Climate Change (IPCC) on lessening the climate alteration with the aim of providing policy makers with the needed policies to lessen the releases of greenhouse gases (GHG) (Jin and Kim 2018).

Innovation could perform a vital function in attaining the sustainability of the environment through the introduction of energy efficient technologies that maintain economic growth without polluting the environment (Haldar and Sethi 2021). Thus, innovation is a key factor in maintaining economic progress and mitigating the climate alteration at the same time (Dauda et al. 2019). Due to the vital errand of technological innovation in ameliorating the environmental quality, the fourth industrial revolution gave great concerns to the environmental quality. This is through introducing technological innovation that depends on cleaner sources of energy; for instance, the smart planning that aims to construct smart residential and commercial buildings which make use of clean energy, and thus reduce the costs associated with inefficient energy use (World Economic Forum 2017).

Innovation could affect environmental quality through different channels. Innovation increases the productivity of capital and labor which results in greater output with the same inputs. Also, innovation causes a shift toward green technologies that protect the environment. Long et al. (2017) found that the economic and environmental behavior of the Korean-owned firms in China is improved by environmental innovation. In addition, innovation improves the market efficiency through government policies that lead to a shift to low-carbon technologies. These policies could include imposing carbon taxes on fossil fuel intensive industries and granting tax credits and subsidies to activities that consume clean energy sources (Mensah et al. 2018).

The objective of this paper is to examine the potential function of technological innovation and clean power in mitigating the ecological footprint in the N-11 countries during the period (1992–2015) by applying panel cointegration analysis with the aim of providing policy makers with the appropriate policies to ameliorate the quality of the environment in those nations. This paper complements the previous literature through several contributions. The first contribution of this paper is to utilize the ecological footprint as a gauge of climate change and environmental deterioration instead of using the carbon dioxide releases or the greenhouse gas releases. The ecological footprint is a consumption-based indicator of environmental sustainability measured in global hectares (gha) that accounts for people’s demand on biological assets and the supply of nature (Global footprint network 2019). The second contribution of this paper is to tackle the function of technological innovation in mitigating the ecological footprint in the N-11 nations. The previous literature that examined the potential role of technological innovation in mitigating environmental degradation is limited and to our best knowledge, those previous studies used the carbon dioxide releases as a gauge of environmental corrosion. The third contribution of this paper is to use the clean energy sources as a determinant of the ecological footprint. The previous limited reviews that utilized the ecological footprint as a gauge of environmental deterioration used the aggregate power consumption as one of the determinants of environmental deterioration without focusing on different power sources. The fourth involvement is related to the countries that this study will be applied to, which are the N-11 nations or the Next 11 nations. The N-11 countries are growing economies that have the opportunity of being some of the largest and greatest economies in the world. The fifth involvement of this study is related to the determinants of environmental deterioration that this study will explore which are clean energy, financial development, technological innovation, and human capital index. The previous literature that explored those determinants is very limited and to our best knowledge, no previous study explored those determinants of environmental deterioration in the N-11 states. Also, to the best of our acquaintance, this is the initial paper that will explore the potential role of clean energy, technological innovation, financial development, and human capital index by utilizing the ecological footprint as a gauge of ecological deterioration.

This study is arranged as demonstrated. Following the introduction, the “The ecological footprint concept” section demonstrates briefly the ecological footprint concept. The “The economic and environmental aspects of the N-11 countries” section presents the economic and the environmental aspects of the N-11 countries. The “Survey of the literature” section displays a summary of the previous literature. The “Model specification and data” section demonstrates the model specification and data. The “Methodology and empirical analysis” section shows the methodology and empirical analysis. The “Conclusion and policy implications**”** section displays the epilogue and policy implications.

## The ecological footprint concept

The ecological footprint is a consumption-based indicator of environmental sustainability measured in global hectares (gha) that accounts for people’s demand on biological assets and the supply of nature. On the demand side, the ecological footprint calculates the amount of ecological assets in terms of the total area of land and water that the population needs to produce its needed goods and to assimilate its residuals and wastes. On the supply side, the ecological footprint gauges the ability of the productive land and water in meeting the nation’s demand to produce the needed consumption goods and absorb the residuals including the emissions of harmful gases. Therefore, if a nation’s entire demand on its ecological assets needed to produce its consumption needs exceeds the nature’s capacity, it is said that the nation runs an ecological deficit. A nation that runs an ecological deficit satisfies its consumption needs either by importing the needed consumption goods that cannot be produced given the available ecological assets or by overusing its supply of natural land and water (for example, over grazing and over fishing) or by polluting the atmosphere through the emissions of harmful gases. On the other hand, if a nation’s supply of ecological assets (country’s biocapacity) exceeds its demand on natural resources, the nation is said to have an ecological reserve. The ecological footprint indicator accounts for six categories of the ecological assets: cropland, fishing grounds, forest products, grazing land, built-up land, and carbon demand on land (Global footprint network 2019). A positive sign of the ecological footprint indicates an increase in environmental degradation, while a negative sign of the ecological footprint indicates a decline in environmental degradation.

## The economic and environmental aspects of the N-11 countries

The Next 11 countries or the N-11 countries have the opportunity of being some of the largest and greatest economies in the world. According to the projections of Goldman Sachs, by the year 2050, two-thirds of the G7 countries size will be shared by the N-11 countries (Raza et al. 2020). The N-11 nations are expected to experience great economic growth such that they are being identified as the next BRICS countries. Due to the expected high economic growth rates in the N-11 nations, the economies of the N-11 countries are expected to compete with existing principal economies and many major markets (Afework et al. 2020).

**Fig. 1:**
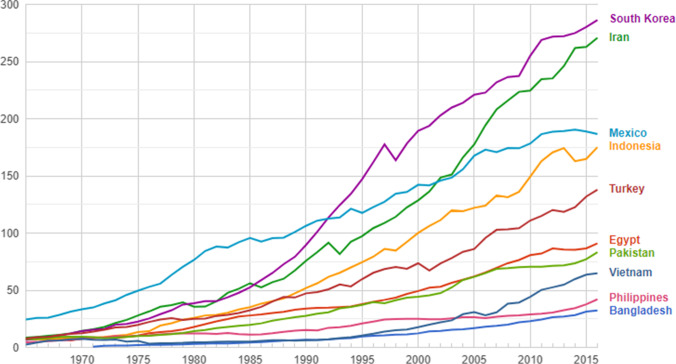
Primary energy consumption (Mtoe) in the N-11 nations over the period 1970–2015. Source: Afework et al. 2020

The N-11 nations aim to promote their economic growth by depending intensively on energy consumption. In 2007, the N-11 countries contributed 9% of the world energy utilization, 30% of the releases of carbon dioxide gases, and 7% of the world economy (Raza et al. 2020). Thus, promoting the economic growth in the N-11 countries is associated with intensive energy demand which resulted in environmental degradation. Figure 1 below shows an expansion in the primary energy consumption in the N-11 nations during the period 1970–2015. This is because the growth in the economies of the N-11 countries is associated with increased energy consumption.

Faced with the environmental concerns, many of the N-11 countries started to formulate policies that support shifting to renewable clean energy sources. For instance, according to the climate scope 2016 report, Mexico’s investments in renewable energy sources reached $4.2 billion in 2016 which represents an expansion in the investments of renewable power sources by 114%. In 2018, many of the N-11 continued their investments in clean power sources with the goal of mitigating the climate change. South Korea invested $5 billion in clean energy sources in 2018, while Mexico invested $3.8 billion in clean energy sources. Moreover, Vietnam invested $3.3 billion in renewable energy sources and Turkey invested $2.2 billion in renewable energy sources (Bloomberg New Energy Finance 2019).

## Survey of the literature

Due to the great concerns of mitigating the climate alteration, many studies investigated the determinants of environmental degradation using different econometric techniques, different variables and applying to different countries and different regions. Those determinants include renewable power consumption, nuclear power utilization and economic growth, among others. Technological innovation can also perform a crucial function in mitigating environmental degradation as technological innovation can lead to a more efficient production process, and thus more efficient usage of natural resources and power (Churchill et al. 2019). Thus, recent studies started to examine the role of technological advancement in mitigating environmental degradation.

On the theoretical side, the theoretical background for technological innovation dates back to the ideas of Josef Schumpeter in the early 1940s who described capitalism as the process of creative destruction in which new technologies and products replace the old ones. Thus, entrepreneurs could have a temporary monopoly power, and as a result, benefit from excess profits for a limited period of time until new products are introduced to the market to replace the existing ones. Schumpeter described three stages through which new technologies enter the market place to replace the existing ones. These three stages are invention, innovation, and diffusion. Invention occurs when a new product is firstly developed. Innovation is achieved when the new product is commercialized in the market place. The stages of invention and innovation are carried out through the R&D. Finally, diffusion is achieved when the new product or technology is adopted by firms or individuals and becomes widely used in many activities (Jaffe et al. 2003). Thus, the technological change process is the collective economic or environmental impact of the three stages. The new growth theory which is recognized also as the endogenous growth theory argues that technological innovation could have a long-run effect on environmental issues including the climate change mitigation issue. Technological change that results from investment in R&D could affect the environmental degradation through introducing energy efficient technologies that lead to changes in the fuel mix and thus reduce the environmental pollution. Thus, technological innovation could help in mitigating the climate change (Ali et al. 2016).

As the ideas of Josef Schumpeter in the early 1940s represent the theoretical background for technological innovation, the green Keynesianism framework represents the theoretical framework for clean energy sources and how they could contribute in mitigating the environmental degradation and achieving ecological sustainability. The green Keynesianism is an expansion to the Keynesian approach that emerged due to the growing concerns of achieving economic growth while maintaining ecological sustainability. The main purpose of the green Keynesianism is to encourage fiscal stimulus programs along with resolving the environmental pollution problems. Thus, the green Keynesianism combines between active macroeconomic policy and environmental goals. This is done through encouraging public investment in clean energy and environmentally friendly technologies that do not emit harmful gases (Cömert 2019).

On the empirical side, this study will categorize the previous literature that explored the determinants of environmental degradation in to four major strands. The first strand of reviews explored the association between economic progress and environmental deterioration. The second strand of reviews examined the relation between different energy sources and environmental deterioration. The third strand of reviews tackled the relation between financial development and the degradation of the environment. The fourth strand of reviews analyzed the relation between technological innovation and environmental deterioration.

### Economic progress and environmental deterioration

For the first group of reviews, many studies investigated the relation between economic progress and environmental deterioration. The results were miscellaneous and indecisive. While some studies found a positive relation between economic progress and environmental deterioration, other studies found a negative or insignificant relation between economic progress and environmental deterioration; as an illustration, the analyses of Azomahou et al. (2006), Poudel et al. (2009), Saboori et al. (2012), Wang (2012), Kasperowicz (2015), Bimonte and Stabile (2017), Lu (2017), Storm and Schröder (2018), Zhang et al. (2019), Jiang et al. (2020) and Zhang (2021).

### Different energy sources and environmental deterioration

Recent empirical studies tackled the relation between different energy sources and environmental deterioration. Some studies found that energy consumption reduces environmental deterioration. For instance, Balogh and Jámbor (2017) for a global sample, Dong et al. (2017) for the BRICS countries, Koengkan and Fuinhas (2017) for South American nations, Bilan et al. (2019) for the European nations, Khan et al. (2020), Busu and Nedelcu (2021) for the European states, Ozcan and Ulucak (2021) for India and Sahoo and Sethi (2021a, b) for 36 developing nations.

Contrastingly, other reviews found that energy consumption does not reduce environmental deterioration. For instance, Menyah and Wolde-Rufael (2010) for the USA, Al-Mulali (2014) for 30 major nuclear-consuming economies, Twumasi (2017) for the USA, and Hasnisah et al. (2019) for the thirteen developing nations in Asia.

### Financial development and environmental deterioration

For the third group of studies, recent scholars considered the potential function of financial development in mitigating environmental degradation. The financial sector has a vital function in promoting economic growth. However, a financial sector could affect the environmental quality while promoting the economic growth of a nation through increasing energy consumption. Thus, it is essential to scrutinize the ecological and environmental aspects of the financial segment (Shahbaz et al., 2018). The relation between financial development and the pollution of the environment was tackled in recent studies and the results were mixed.

For instance, Tamazian et al. (2009) for Brazil, Russia, India, and China, Islam et al. (2013) for Malaysia, Lee et al. (2015) for 25 OECD nations, Saidi and Mbarek (2017) for nineteen emerging nations, Zaidi et al. (2019) for a set of countries, and Guo (2021) for China found that financial development reduces environmental contamination.

In contrast to the previous reviews, Zhang (2011) for China, Shahbaz et al. (2016) for Pakistan, Jiang and Ma (2019) for 155 countries, Bayar and Maxim (2020) for 11 European states, and Nguyen et al. (2021) for Vietnam found that financial development increases the environmental deterioration.

### Technological innovation and environmental deterioration

For the fourth group of studies, limited recent studies analyzed the relation between technological innovation and environmental degradation and the results were mixed. For instance, Apergis et al. (2013) for France, Germany, and the UK, Yii and Geetha (2017) for Malaysia, Shahbaz et al. (2018) for France, Hashmi and Alam (2019) for the OECD nations, and Niu (2021) for China agreed that technological innovation lessens the environmental deterioration.

Contrastingly, Ali et al. (2016) for Malaysia, Jiao et al. (2018) for China, Dauda et al. (2019) for 18 nations, Demir et al. (2020) for Turkey, and Su et al. (2021) for the BRICS found that innovation does not contribute in improving the environmental quality.

To sum up, environmental deterioration has been one of the significant issues that confronted the universe during the past decades. Thus, previous studies as summarized in (Table 1) tackled the sources of environmental degradation with the aim of finding the appropriate policies that could be applied in different countries and different regions to lessen the climate change. The majority of the previous literature used carbon dioxide releases and greenhouse gas releases as gauges of environmental deterioration although the ecological footprint gauge is a more concise indicator of environmental deterioration (Al-Mulali and Ozturk 2015). In addition, no previous study explored the role of technological innovation in mitigating environmental deterioration in the N-11 countries. Thus, this paper will cover the literature gap by examining the potential role of clean power and technological innovation in mitigating the ecological footprint in the N-11 nations during the phase 1992–2015 by applying panel cointegration analysis.

**Table 1:** Empirical studies summary

Study	Countries (period)		Methodology	Variables	Results
**Group one: the relation between economic growth and environmental degradation**					
Saboori et al. (2012)	Malaysia (1980–2009)		Auto Regressive Distributed Lag Approach (ARDL)	CO_2_ Releases Y, Real GDP Per Capita Square	The EKC hypothesis was supported
Kasperowicz (2015)	18 EU member countries (1995–2012)		Error Correction Model (ECM)	CO_2_ Emissions, Energy Consumption, Capital, Total Employment	The EKC hypothesis was supported
Lu (2017)	16 Asian countries (1990–2012)		Panel unit root tests, Panel cointegration estimation, Granger causality Test	Greenhouse Gas Emissions Per Capita, Power Utilization Per Capita, Y, Real GDP Per Capita Square	The EKC hypothesis was supported
Storm and Schröder (2018)	61 countries (1995–2011)		Panel Data Regression	CO_2_ Releases Per Capita, Y, Real GDP Per Capita Square, Population	The EKC hypothesis was supported
Zhang et al. (2019)	China (1996–2015)		ARDL, VECM Granger Causality, Impulse Response, Variance Decomposition	Agricultural CO_2_ Releases, Agricultural Power Utilization, Agricultural Economic Growth	The EKC hypothesis was supported
Jiang et al. (2020)	China and South Korea (2006–2016)		Stimulation Equation Models	Y, SO_2_ Emissions Per Capita, Electricity Consumption, Population, Employment, Share of Manufacturing Industry in GRP	The outcomes reinforced the presence of the EKC hypothesis in the metropolitan areas of both countries
Azomahou et al. (2006)	100 Countries (1960–1996)		Non-Paramteric Data Model	CO_2_ Releases, Per Capita GDP	The EKC hypothesis was not supported
Poudel et al. (2009)	15 Latin American countries (1980–2000)		Semi Parametric Panel Model	CO_2_ Emissions, Per Capita Income, Population, Illiteracy Rate	The EKC premise was not supported
Wang (2012)	98 Countries (1971–2007)		Panel Data Model	CO_2_ Releases, Y, the Square of Per Capita Real GDP	The EKC hypothesis was not supported
Bimonte and Stabile (2017)	Italian regions (1980–2008)		Panel Data Regression	Per Capita Income, Per Capita Supply of New Building Permits, Population	The EKC hypothesis was not supported
Zhang (2021)	China (1971–2014)		Autoregressive distributed lag (ARDL) model	CO2 per capita, Y, annual energy consumption per capita, trade openness, urban population	The EKC hypothesis was not supported
**Group two: the relation between different energy sources and environmental degradation**					
Balogh and Jámbor (2017)		Global sample (1990–2013)	GMM Model	Per Capita CO_2_ Releases, Y, GDP Per Capita Square, Electricity Production from the Nuclear Source, Electricity Production from Coal, Renewable Electricity Output	Nuclear power and renewable power contribute in reducing environmental pollution
Dong et al. (2017)		BRICS countries (1985–2016)	Panel Unit Root Tests, Cointegration, Causality Tests	Per Capita CO_2_ Releases, GDP, Natural Gas Consumption, Renewable Power Utilization	Natural gas and renewable energy consumption contribute in mitigating the climate change
Bilan et al. (2019)		European countries (1995–2015)	Panel Unit Root Tests, Pedroni Cointegration Test, Fully Modified OLS, Dynamic OLS, VECM	Per Capita CO_2_ Emissions, Y, Gross Fixed Capital Formation, Labor Force, Renewable Power Utilization	Renewable energy contributes in mitigating environmental degradation
Koengkan and Fuinhas (2017)		South American countries (1980–2018)	Autoregressive Distributed Lag Approach (ARDL)	CO_2_ Emissions, Real GDP, Renewable Power Consumption, Petroleum Consumption	Renewable power utilization improves the environmental quality
Menyah and Wolde-Rufael (2010)		USA (1960–2007)	Granger Causality Test	CO_2_ Emissions, Real GDP, Renewable Power Utilization, Nuclear Power Consumption	Renewable power utilization consumption has no significant effect on environmental pollution
Al-Mulali (2014)		30 Countries (1990–2010)	Panel Model, Granger Causality Test	CO_2_ Emissions,Y, Electricity Consumption, Domestic Investment, Labor Force, Urbanization, Nuclear Energy Consumption, Fossil Fuel Energy Consumption	Nuclear energy consumption has no effect on environmental pollution
Twumasi (2017)		USA (2009)	Geographic Information System (GIS)	CO_2_ Emissions, Real GDP, Population, Renewable Energy Production	No relation between renewable power production and carbon dioxide releases
Hasnisah et al. (2019)		13 Developing countries (1980–2014)	Panel Cointegration, Fully modified OLS, Dynamic OLS estimators	Per Capita CO_2_ Releases, Real GDP, Real GDP Square, Electricity Utilization from Fossil Fuel Power Sources, Electricity Utilization from Renewable Power Sources	Renewable energy is insignificant in improving the environmental quality
Khan et al. (2020)		Pakistan (1965–2015)	Autoregressive Distributed Lag Approach (ARDL)	Per Capita CO_2_ Emissions, Real GDP, Coal Consumption, Oil Consumption, Natural Gas Consumption	Renewable energy sources contribute in reducing carbon dioxide emissions
Busu and Nedelcu (2021)		European Countries (2009–2019)	Panel Data Multiple Regression Model	CO_2_ Emissions, Renewable Energy, Y, Population, Urbanization, Biofuels, Bioenergy Productivity	Renewable power contributes in lessening carbon dioxide emissions
Ozcan and Ulucak (2021)		India (1971–2018)	Autoregressive Distributed Lag Approach (ARDL)	Y, Nuclear Energy, Population density, CO_2_ Emissions	Nuclear energy improves the ecological quality
Sahoo and Sethi (2021a, b)		36 Developing nations (1990–2016)	Westerlund Cointegration and Dumitrescu and Hurlin Causality test	Ecological Footprint, Y, Renewable Power Utilization, Trade, Globalization, Human Capital	Renewable power lessens the environmental deterioration
**Group three: the relation between financial development and environmental degradation**					
Tamazian et al. (2009)	BRIC countries (1992–2014)		Standard Reduced Form Modeling Approach	CO_2_ Emissions, Y, GDP Per Capita Square, Gross Domestic Expenditure in R&D, FIN	FIN contributes in reducing the environmental degradation
Islam et al. (2013)	Malaysia (1971–2009)		Bounds Testing Approach to Cointegration	Energy Consumption, Y, Population, Financial Development	FIN reduces the emissions of carbon dioxide gases
Lee et al. (2015)	25 OECD countries (1971–2007)		Panel FMOLS and the Cross-Sectional Dependence Regression	CO_2_ Emissions Per Capita, Y, Real GDP Per Capita Square, Energy Use Per Capita, Financial Development	FIN improves the environmental quality
Saidi and Mbarek(2017)	19 Emerging nations (1990–2013)		System- GMM Model	CO_2_ Releases, Income, Income Square, FIN, Urbanization, Overseas Trade	FIN minimizes environmental pollution
Zaidi et al. (2019)	Asia Pacific Economic Cooperation countries (1990–2016)		Westerlund Cointegration Technique, Continuously Updated Bias-Corrected, Continously Updated Fully Modified methods	CO_2_ Releases, Real GDP, Real GDP Square, Power Intensity, FIN, Globalization	FIN could lessen carbon dioxide releases
Zhang (2011)	China (1994–2009)		Cointegration Theory, Granger Causality Test	CO_2_ Releases, Y, FIN, FDI	FIN increases environmental deterioration
Shahbaz et al. (2016)	Pakistan (1985–2014)		Autoregressive Distributed Lag Approach (ARDL)	CO_2_ Emissions, FIN, Power Utilization, Y	FIN in the banking sector could increase carbon dioxide emissions
Jiang and Ma (2019)	155 Nations (1990–2014)		System- GMM Model	CO_2_ Emissions, Financial Development, Trade Openness, Urbanization, Population, Industrial Structure	Financial development could increase environmental pollution in the emerging market and developing countries
Bayar and Maxim (2020)	11 Post-Transition European Countries (1995–2017)		Panel Cointegration, Causality Analyses	CO_2_ Emissions, FIN, Primary Energy Consumption, Real GDP	FIN contributes in increasing the environmental pollution
Guo (2021)	China (1988–2018)		Narayan and Popp unit root test with structural breaks, Maki cointegration, and frequency domain causality test	CO_2_ Emissions, FIN, Renewable Power Electricity, Financial Risk, Human Capital Index	FIN ameliorates the environmental quality
Nguyen et al. (2021)	Vietnam (1986–2019)		Structural Break Unit Root tests, ARDL, and Cointegration Bounds test	CO_2_ Emissions, FDI, Y, FIN, Transportation Capacity	FIN deteriorates the environmental quality
**Group four: the nexus between technological innovation and environmental degradation**					
Apergis et al. (2013)	France, Germany, the UK (1998–2011)		Threshold Autoregressive Model	CO_2_ Emissions, Y, R&D, Oil Prices, Trade Openness	R&D expenditure caused a reduction in carbon dioxide emissions
Yii and Geetha (2017)	Malaysia (1971–2013)		VECM, TYDL Granger Causality Test	CO_2_ Emissions, Y, GDP Square, Electricity Consumption, Energy Price, Technological Innovation	Technological innovation reduces carbon dioxide emissions in the short run
Shahbaz et al. (2018)	France (1955–2016)		Novel SOR Unit Root Test, Bootstrapping Bounds Testing Approach	Per Capita CO_2_ Emissions, Y, Real FDI Per Capita, Energy Consumption, Real Domestic Credit to Private Sector, Population, Public Budget in Power R&D Expenditures	Innovation improves the ecological quality
Hashmi and Alam (2019)	OECD nations (1999–2014)		STIRPRAT Model, Panel Fixed-Effects, Random-Effects, GMM Model	CO_2_ Emissions, Population, Y, Technological Innovation, Environmental Tax Revenue Per Capita	A 1% increase in technological innovation measured by patents reduces carbon emissions by 0.017%
Ali et al. (2016)	Malaysia (1985–2012)		Autoregressive Distributed Lag Approach (ARDL)	CO_2_ Emissions, Y, FIN, Patents Applications	Technological innovation has an insignificant relation with environmental pollution in Malaysia
Jiao et al. (2018)	29 Provinces in China (2000–2013)		Geographic Economic Distance Matrix	Local R&D, Inter-Provincial R&D, FDI	The results were mixed for different Chinese provinces
Dauda et al. (2019)	18 Developing and developed nations(1990–2016)		Fully Modified OLS, Dynamic OLS Estimators	CO_2_ Releases, Real GDP, FDI, Power Consumption, Innovation, Trade Openness	Innovation reduces carbon dioxide releases in G6, while it rises the releases in the MENA countries and the BRICS nations
Demir et al. (2020)	Turkey (1971–2013)		ARDL Bounds Test, Threshold Cointegration Test	CO_2_ Releases, GDP Per Capita, Urban Population, Human Capital Index, Financial Development, Clean Energy, Technological Innovation	There is a reversed U-shape curve between technological innovation and carbon dioxide releases
Su et al. (2021)	BRICS countries (1990–2018)		Panel Regression	Per Capita CO_2_ Emissions, Real GDP, Technological Innovation, Electric Power Consumption, Trade Openness	Technological innovation does not improve the environmental quality
Niu (2021)	China (2009–2018)		Panel Data and Fixed Effects Models	Carbon Emissions, Technological Innovation, Per Capita GDP, Population Size, Industrial Structure, Foreign Trade Dependence	Technological innovation reduces carbon emissions

## Model specification and data

The proposed model for this study is based on the study of Demir et al. (2020). The model is stated as follows:

1 $$\mathrm{EF}=f\left(\mathrm{Y},\mathrm{ URB},\mathrm{ H},\mathrm{ FIN},\mathrm{ CE},\mathrm{ TI}\right)$$

where EF, Y, URB, H, FIN, CE, and TI indicate the ecological footprint, GDP per capita, urban population, human capital index, financial development, clean energy, and technological innovation, respectively. The estimation model is presented below.

2 $${\mathrm{EF}}_{it}={\beta }_{0i}+{\beta }_{1\mathrm{i}} {\mathrm{TI}}_{it}+{\beta }_{2i} {\mathrm{CE}}_{it}+{\beta }_{3i}{\mathrm{FIN}}_{it}+{\beta }_{4i}{\mathrm{H}}_{it}+{\beta }_{5i} {\mathrm{Y}}_{it}+{\beta }_{6i }{\mathrm{URB}}_{it}+{\varepsilon }_{it}$$

where *i* denotes each cross section (countries) in this study and *t* denotes the time frame. The time frame in this study covers the period (1992–2015). *β* refers to the slope coefficient of the corresponding variable. It shows that a one unit rise in the explanatory variable will increase (decrease) the ecological footprint by *β* units, holding other variables constant. *ε*_*it*_ denotes the estimation residual. Table 2 below demonstrates the variables utilized in this study, their definitions, units of measurement, and data sources.

**Table 2:** Variables, definitions and data sources

Variables	Measurement units and definition	Data sources
EF	The ecological footprint is a consumption based indicator of environmental sustainability measured in global hectares (gha). The ecological footprint accounts for people’s demand on biological assets and the supply of nature	Global Footprint Network
Y	Real GDP per capita assessed in constant 2010 US dollar	World Bank Development Indicators (WDI)
CE	Clean energy measured by renewable power consumption (percentage of aggregate final power consumption)	WDI
FIN	Financial development gauged by domestic credit to private sector (percentage of GDP)	WDI
URB	Urban population measured as % of the total	WDI
H	Human capital index is calculated by relying on the years of schooling and returns to education	Penn World Table 9.^1^,^2019^(pwt 9.1)
TI	Technological innovation gauged by the sum of resident and non-resident patent applications	WDI

Table 3 below demonstrates the variables descriptive statistics during the time period of the study (1992–2015). The time frame (1992–2015) is chosen because this is the longest series available; in addition, this period witnessed the exacerbation of the environmental degradation problem in those countries. As shown in the variables descriptive statistics, the mean shows the average value for each of the variables. For instance, the mean value of the ecological footprint variable is 180,253,488.2. The median shows the middle value for each of the variables. The standard deviation tells us the deviation from the sample mean with respect to each of the variables. According to the descriptive statistics displayed in Table 3 below, Indonesia has the highest ecological footprint across the 9 countries (429,070,148.9 global hectares (gha) in 2014), while Vietnam has the lowest ecological footprint across countries (51,752,016 global hectares (gha) in 1992). South Korea has the maximum GDP per capita across nations (26,063.71 US dollar in 2015), while Bangladesh has the lowest GDP per capita across countries (428.6610 US dollar in 1992). With respect to the clean energy variable, Vietnam has the highest value (74.70197 in 1992), while South Korea has the lowest value (0.441575 in 1994). With respect to the financial development variable, South Korea has the highest value (148.3405 in 2008), while Mexico has the lowest value (12.87772 in 2001). South Korea has the highest urban population across countries (81.93600 in 2010), while Bangladesh has the lowest urban population across countries (20.61000 in 1992). South Korea has the highest human capital index across countries (3.626602 in 2015), while Pakistan has the lowest human capital index across countries (1.393996 in 1992). Regarding the technological innovation variable, South Korea has the highest value (213,694 in 2015), while Vietnam has the lowest value (83 in 1992).

**Table 3:** The variables descriptive statistics

	**EF**	**Y**	**CE**	**FIN**	**URB**	**H**	**TI**
Mean	180,253,488.2	5088.905	29.57496	41.51572	49.21134	2.255319	17,727.76
Median	149,098,442.5	2124.386	32.32325	29.16280	44.75250	2.238592	2440.000
Maximum	429,070,148.9	26,063.71	74.70197	148.3405	81.93600	3.626602	213,694.0
Minimum	51,752,016	428.6610	0.441575	12.87772	20.61000	1.393996	83.00000
Std. Dev	93,529,898	5909.128	20.46024	30.78190	19.54882	0.489231	44,190.62
Skewness	0.630805	1.665013	0.120126	1.913387	0.380729	0.746096	3.092452
Kurtosis	2.272098	5.283161	1.744209	6.010771	1.757911	3.336445	11.52207
Jarque–Bera	19.09352	146.7171	14.71260	212.3925	19.10344	21.05847	979.4284
Probability	0.000071	0.000000	0.000639	0.000000	0.000071	0.000027	0.000000

## Methodology and empirical analysis

### Methodology

Based on the studies of Jin and Kim (2018), Saidi and Mbarek (2016), and Al-Mulali (2014), this paper will firstly perform two-unit root tests to check the stationary properties of the variables. The two-unit root tests are the Fisher-Augmented Dickey Fuller (ADF) unit root test, and the Phillips and Perron (PP) unit root test. The null hypothesis of both of them is the presence of unit root. Secondly, this paper will conduct the Pedroni co-integration test and Kao residual co-integration test to explore long run dynamic relation among the variables. Thirdly, this paper will adopt the Fully Modified OLS (FMOLS) and Dynamic OLS (DOLS) estimators with the aim of analyzing the cointegrating vector between the cointegrated variables proved by the cointegration analysis. Fourthly, this paper will investigate the Granger causality through the Vector Error Correction Model (VECM) that is going to be applied to estimate the Granger causality if the cointegration relationship is proven between the variables, while in case of no long-run relation; the Vector Autoregressive (VAR) model will be applied.

### Empirical analysis and results

#### Panel unit root tests

This paper performed the ADF and the PP tests to test stationarity. Both tests are established upon the Chi-square (Al-Mulali 2014). The null hypothesis of both of them is the existence of unit root which signifies the non-stationarity of the series.

Table 4 below demonstrates the panel unit root tests outcomes. The lag length choice is established upon Schwarz Information Criterion (SIC) and is selected automatically. In compliance with the results of the ADF test and the PP test, the null hypothesis of the ecological footprint (EF) variable, the per capita GDP (Y) variable, the clean energy (CE) variable, the financial development (FIN) variable, the urban population (URB) variable, and the technological innovation (TI) variable cannot be rejected at 1% significance level. This indicates that at level, the six series are non-stationary. By applying the first difference for the ecological footprint (EF) variable, the GDP per capita (Y) variable, the clean energy (CE) variable, the financial development (FIN) variable, and the technological innovation (TI) variable, the null hypothesis cannot be accepted at 1% level of significance. The urban population (URB) variable becomes stationary after taking the second difference. Moreover, the null hypothesis of the human capital index (H) variable can be rejected at 1% significance level which signifies that at level, the human capital index (H) series is stationary.

**Table 4:** Panel unit root test outcomes at level, after applying the first difference and after applying the second difference

Variables	Panel unit root test	Level statistic (P-value)	First difference statistic (P-value)	Second difference statistic (P-value)
Ecological Footprint (EF)	ADF	23.1780 (0.1838)	114.999 (0.0000)^b^	-
	PP	23.1235 (0.1859)	183.921 (0.0000)^b^	-
GDP per capita (Y)	ADF	18.5693 (0.4188)	76.8279 (0.0000)^b^	-
	PP	9.86380 (0.9363)	88.2778 (0.0000)^b^	-
Clean Energy (CE)	ADF	33.7263 (0.0136)	94.9874 (0.0000)^b^	-
	**PP**	16.8862 (0.5309)	152.822 (0.0000)^b^	-
Financial Development (FIN)	ADF	16.0605 (0.5883)	60.4374 (0.0000)^b^	-
	PP	6.04127 (0.9960)	85.8447 (0.0000)^b^	-
Urban Population (URB)	ADF	22.5265 (0.1270)	23.7160 (0.0959)	54.9057 (0.0000)^b^
	PP	19.3856 (0.2492)	6.36599 (0.9836)	36.9240 (0.0021)^b^
Human Capital Index (H)	ADF	279.666 (0.0000)^b^	-	-
	PP	60.5485 (0.0000)^b^	-	-
Technological Innovation (TI)	ADF	20.0012 (0.3328)	78.7553 (0.0000)^b^	-
	PP	12.4933 (0.8208)	343.491 (0.0000)^b^	-

^b^ Signifies the refusal of the null hypothesis at 1% level of significance

Thus, the ecological footprint (EF) variable, the GDP per capita (Y) variable, the clean energy (CE) variable, the financial development (FIN) variable, and the technological innovation (TI) variable follow the I(1) process. The urban population (URB) variable follows the I(2) process. The human capital index (H) variable follows the I(0) process. By considering these results, we will continue with performing the cointegration tests to explore the long-run relation among the variables that pursue the I(1) process.

#### Panel cointegration tests

This paper carried out the Pedroni cointegration test to evaluate the association in the long-run among the non-stationary variables that pursue the I(1) process. To prove the reliability of the Pedroni cointegration test results, this paper performed the test of Kao cointegration. The null hypothesis of the two tests is the non-existence of cointegration between variables (Al-Mulali and Ozturk 2015). The lag length choice is established upon the SIC and is selected automatically.

Both the Pedroni and the Kao tests are Engle and Granger–based cointegration tests that depend on the assessment of residuals. In case not rejecting the null hypothesis, then the residuals will be I(1) which reveals the absence of a long-run association between the variables. If the null hypothesis is rejected, then the residuals will be I(0) which indicates a long-run association among the variables. The Pedroni cointegration test derives seven test statistics from the estimated residuals. Four of the seven test statistics are established upon within-dimension statistics. The other three test statistics are established upon between-dimension statistics (Jin and Kim, 2018).

Tables 5 and 6 below show the outcomes of the two tests. Both tests proved the existence of cointegration between the ecological footprint (EF) variable, the clean energy (CE) variable, the GDP per capita (Y) variable, the financial development (FIN) variable, and technological innovation (TI) variable.

**Table 5:** Pedroni cointegration test outcomes. Alternative hypothesis: common AR coefs. (within-dimension)

	Statistic	Prob	Weighted statistic	Prob
Panel v-Statistic	− 0.353463	0.6381	1.605034	0.0542 ^f^
Panel rho-Statistic	− 0.768657	0.2210	− 0.399706	0.3447
Panel PP-Statistic	− 3.690251	0.0001 ^b^	− 3.143656	0.0008 ^b^
Panel ADF-Statistic	− 3.931148	0.0000 ^b^	− 4.586214	0.0000 ^b^
Alternative hypothesis: individual AR coefs. (between-dimension)				
	Statistic			Prob
Group rho-Statistic	0.686527			0.7538
Group PP-Statistic	− 3.238641			0.0006 ^b^
Group ADF-Statistic	− 5.043014			0.0000 ^b^

^b^ and ^f^ signify the refusal of the null hypothesis at 1% and 10% level of significance, respectively

**Table 6:** Kao cointegration test outcomes

	**t-Statistic**	**Prob**
ADF	− 1.501115	0.0667 ^f^
Residual variance	2.27E + 14	
HAC variance	2.22E + 14	

^f^ Signifies the refusal of the null hypothesis at 10% significance level

## Panel long-run estimator

After carrying out the tests of cointegration, a long-run estimator is adapted to assess the long-run association among the study variables. Running a regression that involves those I(1) variables will be associated with the spurious problem which will lead to misleading results through showing a significant relationship between unrelated series (Al-Mulali 2014). To overcome this problem, the FMOLS and the DOLS estimators have been lately employed in the literature due to their effectiveness in disposing the issues of endogeneity in the explanatory variables and the serial correlations in the error terms. Moreover, the variables possess large sample properties. The FMOLS estimator uses the non-parametric method to eliminate the difficulties of endogeneity and autocorrelation. On the other hand, the DOLS estimator uses the parametric method, and lags and leads of the regressors to abolish the problems of endogeneity and autocorrelation (Dogan and Seker 2016).

Tables 7 and 8 below show the outcomes of the FMOLS and the DOLS estimators. All the coefficients estimated are significant at 1% significance level excluding the financial development (FIN) variable which is insignificant according to the DOLS estimator. The coefficients signs are consistent. Whereas the GDP per capita (Y) variable has a positive relation with the ecological footprint, the clean energy (CE) variable, the financial development (FIN) variable, and the technological innovation (TI) variable have a negative relation with the ecological footprint. In compliance with the results FMOLS and the DOLS, a one-unit rise in the per capita GDP will expand the environmental degradation measured by the ecological footprint by 18,549 units and 12,868 units, respectively. Conversely, a one-unit rise in the clean power will reduce the environmental degradation by 3,479,098 units and 2,302,024 units, respectively. A one-unit increase in the technological innovation will alleviate the environmental degradation by 703 units and 453 units, respectively. The results of the FMOLS show that a one-unit rise in the financial development will reduce the environmental deterioration by 449,091 units. Thus, clean energy and technological innovation can contribute in mitigating the environmental deterioration in the long run. Conversely, economic activity will be linked with environmental deterioration in the long run.

**Table 7:** Outcomes of the panel FMOLS estimator

Variables	Coefficient	Std. Error	t-Statistic	Prob
Y	18,548.57	0.010446	1,775,672	0.0000^b^
CE	− 3,479,098	0.018703	− 1.86E + 08	0.0000^b^
FIN	− 449,090.6	0.022593	− 19,877,146	0.0000^b^
TI	− 702.5748	0.024209	− 29,021.43	0.0000^b^
R-squared 0.958245

**Table 8:** Outcomes of the panel DOLS estimator

Variables	Coefficient	Std. Error	t-Statistic	Prob
Y	12,867.60	1740.893	7.391379	0.0000^b^
CE	− 2,302,024	341,565.1	− 6.739634	0.0000^b^
FIN	− 62,736.52	140,397.6	− 0.446849	0.6569
TI	− 452.5241	141.6578	− 3.194488	0.0024^b^
R-squared 0.989991				

The leads and lag specification are established upon SIC and is selected automatically. b signifies the refusal of the null hypothesis at 1 percent significance level

These outcomes are in compliance with the economic circumstances of the Next 11 nations. A positive sign of per capita GDP with environmental deterioration is consistent with the economic circumstances of the Next 11 nations. The Next 11 nations rely intensively on energy consumption in fostering their economic progress without considering the efficiency issues in energy utilization. Thus, fostering the economic progress is associated with deteriorating the environment in the Next 11 nations. Moreover, a negative sign of clean power and innovation with environmental deterioration is consistent with the economic circumstances of the Next 11 countries. This is because shifting toward the deployment of clean power sources and innovation that consolidates the deployment of renewable power sources contribute in lessening the environmental deterioration. Additionally, these outcomes are consistent with the preceding literature. In compliance with the previous literature, nations that tend to consolidate their economic growth without considering the efficiency issues suffer from environmental deterioration (For instance, the studies of Lu (2017), Storm and Schröder (2018), Zhang et al. (2019) and Jiang et al. (2020)). Also, it is consistent with the preceding literature that shifting toward the deployment of clean power sources ameliorates the quality of the environment (For instance, the studies of Katircioglu (2015), Balogh and Jámbor (2017), Dong et al. (2017), Bilan et al. (2019) and Chandio et al. (2021)).

## Panel Granger causality

This paper investigated Granger causality through the VECM. The VECM allows us to assess the short- and long-run dynamics of the series that are integrated of order one. The VECM is applied through a two-phase procedure. The first phase is assessing the long-run parameters from the cointegrating equation, and then deriving the residuals. Those residuals are treated as lagged error correction term (ECT). The second phase is estimating the dynamic VECM. The dynamic VECM is stated as demonstrated.

3 $$\begin{array}{c}{\Delta \mathrm{EF}}_{it}={\beta }_{1i}+{\sum }_{j= 1}^{\lambda } {\beta }_{11i j} {\Delta \mathrm{EF}}_{it-j}+{\sum }_{j= 1}^{\lambda } {\beta }_{12i j} \Delta {Y}_{it-j}+{\sum }_{j= 1}^{\lambda } {\beta }_{13i j} \Delta {\mathrm{CE}}_{it-j} \\ +{\sum }_{j= 1}^{\lambda }{\beta }_{14i j} \Delta {\mathrm{FIN}}_{it-j}+ {\sum }_{j= 1}^{\lambda } {\beta }_{15i j} \Delta {\mathrm{TI}}_{it-j}+ {\uptheta }_{1\mathrm{i}} {\mathrm{ECT}}_{it-1}+{\varepsilon }_{1it}\end{array}$$

4 $$\begin{array}{c}\Delta {\mathrm{Y}}_{it}={\beta }_{2i}+{\sum }_{j= 1}^{\lambda } {\beta }_{21i j} \Delta {\mathrm{EF}}_{it-j}+{\sum }_{j= 1}^{\lambda } {\beta }_{22ij} \Delta {Y}_{it-j}+{\sum }_{j= 1}^{\lambda }{ \beta }_{23i j} \Delta {\mathrm{CE}}_{it-j} \\ +{\sum }_{j= 1}^{\lambda }{\beta }_{24i j} \Delta {\mathrm{FIN}}_{it-j}+ {\sum }_{j= 1}^{\lambda } {\beta }_{25i j} \Delta {\mathrm{TI}}_{it-j}+ {\uptheta }_{2i} {\mathrm{ECT}}_{it-1}+{\varepsilon }_{2it}\end{array}$$

5 $$\begin{array}{c}\Delta {\mathrm{CE}}_{it}={\beta }_{3i}+{\sum }_{j= 1}^{\lambda } {\beta }_{31i j} \Delta {\mathrm{EF}}_{it-j}+{\sum }_{j= 1}^{\lambda } {\beta }_{32ij} \Delta {Y}_{it-j}+{\sum }_{j= 1}^{\lambda }{ \beta }_{33i j} \Delta {\mathrm{CE}}_{it-j} \\ +{\sum }_{j= 1}^{\lambda }{\beta }_{34i j} \Delta {\mathrm{FIN}}_{it-j}+ {\sum }_{j= 1}^{\lambda } {\beta }_{35i j} \Delta {\mathrm{TI}}_{it-j}+ {\uptheta }_{3i} {\mathrm{ECT}}_{it-1}+{\varepsilon }_{3it}\end{array}$$

6 $$\begin{array}{c}\Delta {\mathrm{FIN}}_{it}={\beta }_{4i}+{\sum }_{j= 1}^{\lambda } {\beta }_{41i j} \Delta {\mathrm{EF}}_{it-j}+{\sum }_{j= 1}^{\lambda } {\beta }_{42ij} \Delta {Y}_{it-j}+{\sum }_{j= 1}^{\lambda }{ \beta }_{43i j} \Delta {\mathrm{CE}}_{it-j} \\ +{\sum }_{j= 1}^{\lambda }{\beta }_{44i j} \Delta {\mathrm{FIN}}_{it-j}+ {\sum }_{j= 1}^{\lambda } {\beta }_{45i j} \Delta {\mathrm{TI}}_{it-j}+ {\theta }_{4i} {\mathrm{ECT}}_{it-1}+{\varepsilon }_{4it}\end{array}$$

7 $$\begin{array}{c}\Delta {\mathrm{TI}}_{it}={\beta }_{5i}+{\sum }_{j= 1}^{\lambda } {\beta }_{51i j} \Delta {\mathrm{EF}}_{it-j}+{\sum }_{j= 1}^{\lambda } {\beta }_{52ij} \Delta {Y}_{it-j}+{\sum }_{j= 1}^{\lambda }{ \beta }_{53i j} \Delta {\mathrm{CE}}_{it-j} \\ +{\sum }_{j= 1}^{\lambda }{\beta }_{54i j} \Delta {\mathrm{FIN}}_{it-j}+ {\sum }_{j= 1}^{\lambda } {\beta }_{55i j} \Delta {\mathrm{TI}}_{it-j}+ {\uptheta }_{5i} {\mathrm{ECT}}_{it-1}+{\varepsilon }_{5it}\end{array}$$

where ∆ refers to the first difference operator, λ denotes the lag length, *β*_*i*_ refers to the nation fixed effect and ECT denotes the lagged error correction term. The error correction term refers to the truth that the short-run dynamics of the dependent variable is influenced by the last period deviation from the long-run equilibrium. Moreover, Ө is the error correction term (ECT) coefficient (Elshimy and El-Aasar 2019).

The VECM enables us to assess the short- and the long-run dynamics of the cointegrated series. The significance of the variables lagged differences is examined to determine the short-run causal relations. The significance of ECT is examined to determine the long-run causal relations. A negative and statistically significant error correction term signifies the occurrence of a long-run causal relation (Elshimy and El-Aasar 2019).

Table 9 below displays the outcomes of the VECM Granger-causality. The ideal lag length is one established upon Schwarz Information Criterion (SIC). For the short-run causality, with respect to Eq. (3) and Eq. (4), the short-run causal relation is not found. With respect to Eq. (5), there exists short-run causal link from financial development to clean power. With respect to Eq. (6) and Eq. (7), there exists short-run bidirectional causal relation between financial development and technological innovation.

**Table 9:** Outcomes of the VECM Granger-causality

Regressand variable	Short run					Long run
	∆EF	∆Y	∆CE	∆FIN	∆TI	ECT
∆ EF	**–**	[0.902973]	[− 0.039695]	[− 0.047415]	[0.121824]	[0.756544]
		(0.3668)	(0.9683)	(0.9622)	(0.9031)	(0.4495)
∆ Y	[− 0.438849]	**–**	[0.757602]	[0.957865]	[0.474433]	[− 6.040587]
	(0.6609)		(0.4489)	(0.3384)	(0.6353)	(0.0000)^b^
∆ CE	[0.420090]	[0.148018]	**–**	[− 2.662980]	[− 0.439899]	[− 3.736330]
	(0.6745)	(0.8824)		(0.0079)^b^	(0.6601)	(0.0002)^b^
∆ FIN	[0.372387]	[0.995712]	[− 0.786852]	**–**	[2.572420]	[0.673936]
	(0.7097)	(0.3197)	(0.4316)		(0.0103)^d^	(0.5005)
∆ TI	[− 0.421043]	[1.043578]	[− 0.613968]	[− 2.060620]	**–**	[− 2.983861]
	(0.6738)	(0.2970)	(0.5394)	(0.0396)^d^		(0.0029)^b^

The *t*-statistics are written in [], while the *P*-values are written in (). ^b^ and ^d^ signify the statistical significance at 1% and 5%, respectively

Regarding the long-run causality, in view of Eq. (3) and Eq. (6), there is no long-run causality. In view of Eq. (4), there exists a long-run causal relation from the ecological footprint, the clean energy, the financial development, and the technological innovation to the GDP per capita. In view of Eq. (5), a long-run causal relation exists from the ecological footprint, the GDP per capita, the financial development, and the technological innovation to clean energy. In view of Eq. (7), a long-run causal relation exists from the ecological footprint, the GDP per capita, the clean energy and the financial development to technological innovation. Thus, a long-run unidirectional relation exists from the ecological footprint to GDP per capita, clean energy, and technological innovation.

**Fig. 2:**
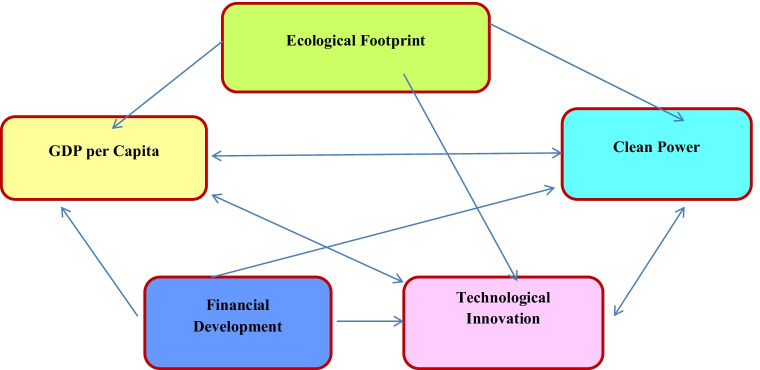
The long-run causal relation between the ecological footprint, the GDP per capita, the clean power, the financial development and the technological innovation

The outcomes of the VECM Granger-causality are consistent with the economic circumstances of the Next 11 countries as environmental deterioration faced by those countries leads to shifting toward clean power sources and innovation in the long-run. Moreover, clean power and innovation can contribute in fostering the economic progress of the Next 11 nations without polluting the environment which is consistent with the preceding studies (For instance, the studies of Santra (2017), Mensah et al. (2018), Hashmi and Alam (2019), and Sahoo and Sethi (2021a, b)). This is because renewable power sources satisfy the nations’ energy needs which foster the economic progress without deteriorating the environment. Additionally, financial development—with appropriate policies—can enhance the utilization of clean power sources which is also consistent with the previous literature (For example, the studies of Lee et al. (2015), Dogan and Seker (2016), Saidi and Mbarek (2017) and Zaidi et al. (2019)). Figure 2 below displays the long-run and causal relations between the ecological footprint, the per capita GDP, the clean power, the financial development, and the technological innovation.

## Conclusion and policy implications

This paper examined the potential role of clean power and innovation in mitigating the ecological footprint in the Next 11 countries. With the purpose of investigating the potential role of clean power and innovation in alleviating the ecological footprint, this paper applied panel cointegration analysis. The Pedroni cointegration test and the Kao cointegration test proved the occurrence of long-run relation among the ecological footprint (EF) variable, the clean energy (CE) variable, the per capita GDP (Y) variable, the financial development (FIN) variable, and the technological innovation (TI) variable. The VECM enables us to assess both the short- and the long-run dynamics of the cointegrated series. Regarding the short-run causal relations, there exists short-run causal link from financial development to clean power. Moreover, there exists short-run bidirectional causal relation between financial development and technological innovation. Regarding the long-run dynamics, there exists a long-run causal relation from the ecological footprint, the clean energy, the financial development, and the technological innovation to the per capita GDP. Additionally, there exists a long-run causal relation from the ecological footprint, the per capita GDP, the financial development, and the technological innovation to clean energy. Moreover, a long-run causal relation exists from the ecological footprint, the GDP per capita, the clean power, and the financial development to technological innovation.

The outcomes of the VECM imply that the environmental degradation (measured by the ecological footprint variable) faced by those nations leads to shifting toward clean energy sources and technological innovation in the long-run. Technological innovation enhances improving the energy efficiency and shifting toward green technologies that are environmentally friendly (Ibrahiem 2020). Thus, the N-11 countries are in need to design policies that enhance shifting toward environmentally friendly energy sources. These policies may include reducing the portion of fossil fuel power sources and expanding the portion of clean power sources in the energy consumption. Subsidies and low-interest loans may be given to the producers of clean power sources. On the other hand, taxes may be imposed on the producers of fossil fuel energy sources with the aim of increasing the portion of renewable power sources in the fuel mix. Additionally, the nation’s governments may construct electricity generators that enhance the utilization of renewable power sources (Cheng et al. 2019). Moreover, the nation’s government may import electric vehicles that are environmentally friendly. Also, there is a need for R&D subsidies that enhance the expansion and usage of clean power sources.

Clean power sources and technological innovation that improves energy efficiency can be utilized as tools of fostering the economic progress in the long run. Thus, clean energy sources can replace the fossil fuel energy sources which promote the economic progress in the Next 11 nations without deteriorating the environment. This is consistent with the policies of the international organizations which give great concern to the matter of lessening the climate change with the aim of shifting to cleaner and sustainable sources of power.

Finally, financial development can promote economic progress in the Next 11 nations without causing environmental contamination in the long-run. Financial development enhances the productivity of the inputs and fosters capital accumulation which promotes the economic growth. Financial development can be associated with environmental degradation if it promoted the energy intensive industries (Ibrahiem, 2020). For example, financial organizations can provide credit facilities to their customers to stimulate investments in projects that can be associated with the emissions of harmful gases. Moreover, loan facilities can promote the purchase of automobiles, electrical devices and equipment that result in the emissions of harmful gases and environmental degradation (Ganda 2019). However, with appropriate nations’ policies that aim to mitigate the climate changes, financial development can be used as a tool of mitigating the environmental degradation. For instance, loan facilities can be given to projects that depend on clean energy sources instead of projects that depend intensively on fossil fuel energy sources which promote the economic growth of the nation without polluting the environment. Thus, with appropriate nations’ policies, financial development can be used as a method of ameliorating the environmental quality.

### Scope for future research

Further research could focus on the role of nuclear power in enhancing the development and improving the environmental quality of the developing nations.

## Data Availability

Data is available at World Bank and the link is: https://data.worldbank.org/data-catalog/world-development-indicators

## Appendix

### List of countries

- Bangladesh
- Egypt
- Indonesia
- Iran
- Mexico
- Nigeria
- Pakistan
- The Philippines
- Turkey
- South Korea
- Vietnam
